# A Targeted DNAzyme-Nanocomposite Probe Equipped with Built-in Zn^2+^ Arsenal for Combined Treatment of Gene Regulation and Drug Delivery

**DOI:** 10.1038/srep22737

**Published:** 2016-03-09

**Authors:** Zhi-Mei He, Peng-Hui Zhang, Xin Li, Jian-Rong Zhang, Jun-Jie Zhu

**Affiliations:** 1 State Key Laboratory of Analytical Chemistry for Life Science and Collaborative Innovation Center of Chemistry for Life Sciences, School of Chemistry & Chemical Engineering, Nanjing University, Nanjing 210093, P.R. China; 2 State Key Laboratory of Pharmaceutical Biotechnology, School of Life Science, Nanjing University, Nanjing 210023, P.R. China; 3 School of Chemistry and Life Science, Nanjing University Jinling College, Nanjing 210089, China

## Abstract

As catalytic nucleic acids, DNAzymes have been extensively used in the design of sensing platforms. However, their potentials as intelligent drug carriers for responsive drug release in gene therapy and chemotherapy were rarely explored. Herein, we report a dual-functional probe composed of gold nanoparticles (GNPs), catalytic Zn^2+^-dependent DNAzyme, anticancer drug doxorubicin (Dox), targeted AS1411 aptamer and acid-decomposable ZnO quantum dots (ZnO QDs) to achieve intracellular gene regulation and drug delivery in a controlled manner. By means of aptamer-guided targeting and receptor-mediated endocytosis, the probes were specifically internalized into the HeLa cells and trapped in the acidic endo-/lysosomes, where the ZnO QDs as the built-in Zn^2+^ arsenal were promptly dissolved to offer Zn^2+^, leading to the activation of DNAzyme to cleave the substrate strands, and subsequent drug release. Meanwhile, as designed, one part of the cleaved substrate, hybridized with the overexpressed miR-21 in the target cells, thereby declining its intracellular level. Taken together, the down-regulation of miR-21 has a synergistic effect with Dox to efficiently eradicate the cancer cells. Thus, the favorable biocompatibility, cancer cell specificity and combined treatment make the probe promising for therapy of multidrug-resistant cancer and *in vivo* application.

MicroRNAs (miRNAs) are a category of small noncoding RNA molecules of ~22 nucleotides that regulate gene expression in a wide range of physiological processes including cellular development^1^, differentiation^2^, proliferation^3^, apoptosis^4^, hematopoiesis^5^, etc. In recent years, accumulated evidence has showed that aberrant expression of miRNAs is closely correlated with the initiation, development, and metastasis of various cancers, where they can function as tumor suppressors or oncogenes, highlighting their significance in human cancer^6^. miR-21, an oncogenic miRNA, has been found overexpressed in various cancers^7^,^8^. The inhibition of miR-21 expression by delivering antisense sequence can down-regulate antiapoptotic genes such as Bcl-2, resulting in the decrease of cell proliferation and increase of apoptosis^9^. Thus, chemical tools developed for either detection or regulation of endogenous miR-21 may provide great potential for cancer therapy.

Deoxyribozymes, denoted as DNAzymes, are a class of artificial single-stranded DNA molecules with catalytic activities^10^,^11^,^12^, whose functions are performed by recruiting cofactors such as metal ions or organic molecules. Particularly, many metal-dependent DNAzymes have high affinities for specific metal ions. In the presence of corresponding metal ions, the specific DNAzymes fold into compact structures to activate catalytic function of cleavage^13^, making themselves widely applicable in the detection of metal ions such as Pb^2+^ 14,15,16,17, Cu^2+^ 18,19, Zn^2+^ 20 and UO_2_^2+^ 21,22. Compared with RNA- or protein-based enzymes, DNAzymes are more stable against nuclease degradation and less susceptible to hydrolysis, which endow them with unique applicability in the fields of biochemistry and pharmacology^23^,^24^. However, in the bio-applications^25^,^26^ other than aforementioned metal ion sensors, switching on the DNAzyme activity relies heavily on the introduction of external metal ions. In this regard, incorporating a self-generation source of metal ions may provide the stimulus for initiating the catalytic cascades.

ZnO QDs have been reported as gatekeepers to cap mesoporous silica nanoparticles (MSNs) for pH-triggered drug release, because they decompose at pH < 5.5 while keep stable at pH 7.4^27^. In consideration of the acidic environment inside lysosomes or endosomes (pH 4.5–6.5)^28^,^29^, ZnO QDs are suitable as potential Zn^2+^ providers for intracellular applications due to their pH-sensitivity. Inspired by this feature, we designed a smart probe for controlled gene regulation and drug delivery based on the Zn^2+^-dependent DNAzyme.

In this system, doxorubicin (Dox) encapsulated and antisense RNA-integrated DNAzyme/substrate bioconjugate was grafted on a ZnO QDs-contained probe, which was targeted to cancer cells to achieve synergistic effect between anti-cancer drug and gene silencing agent. The assembly process for the probe was illustrated in Fig. 1. Firstly, the Zn^2+^-dependent ligation DNAzyme and AS1411 aptamer were assembled onto the gold nanoparticles (GNPs) via Au-S bonding, followed by the hybridization between the DNAzyme and its substrate strand. Secondly, Dox as a model drug was intercalated in the double-stranded GC pairs. Finally, positively charged ZnO QDs as potential Zn^2+^ providers were electrostatically adsorbed onto GNPs to fabricate the intracellular probe (GNPs-ES-Dox/ZnO). In the presence of AS1411 aptamer, the probes were actively transported into HeLa cells via the recognition between aptamer and nucleolin overexpressed on the plasma membrane of most cancer cells^30^. Upon internalization into the acidic organelles of HeLa cells, the cleavage activity of DNAzyme was switched on by Zn^2+^ ions self-generated from the disintegration of ZnO QDs, which triggered the subsequent release of Dox. Simultaneously, the cleaved subunit of the substrate strand which was designed to contain anti-miR-21, tightly bound to the endogenous miR-21 to reduce its intracellular level, consequently contributing to the inhibition of cell proliferation and activation of cell apoptosis. The synergistic combination of controlled Dox release and down-regulation of miR-21 can efficiently eradicate cancer cells, thus offering great promise for treatment of multidrug-resistant cancer. Furthermore, through elaborate molecular design, the approach may be readily extended to other gene regulation by substituting the anti-miR-21 sequence with other anti-miRNA.

## Results and Discussion

### Characterization of GNPs-ES-Dox/ZnO

**Figure 1:**
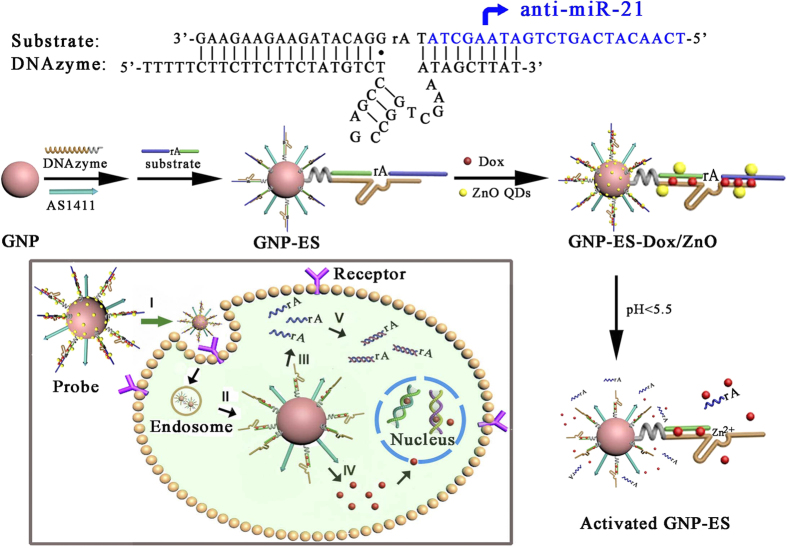
Schematic illustration of the nanoprobe for controlled Dox release and gene regulation. Stage: I, aptamer-targeted recognition and receptor-mediated endocytosis; II, endosomal escape; III, detachment of anti-miR-21 containing subunits from the assembly; IV, pH-triggered Dox release; V, inhibition of endogenous miR-21.

**Figure 2:**
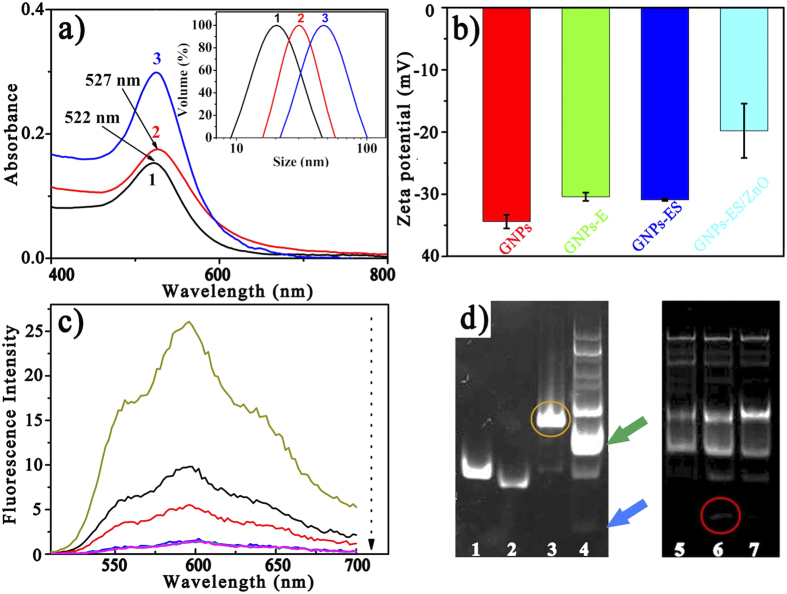
(**a**) UV-Vis spectra of (1) GNPs, (2) GNPs-E and (3) GNPs-ES (inset: Hydrodynamic size distributions of the corresponding nanoparticles). (**b**) The changes of zeta potential during the preparation of GNPs-ES/ZnO nanocomplex. Error bars indicate s.d. (n = 3). (**c**) Photoluminescence spectra of Dox solution (5.5 mg mL^−1^) with increasing mole ratios of hybridized complex of DNAzyme and substrate in 20 mM HEPES (pH 7.4, 100 mM NaCl) (top to bottom: 0, 0.1, 0.2, 0.5, 0.8, 1 μM). (**d**) Determination of DNAzyme cleavage by polyacrylamide gel electrophoresis. Samples: (1) E; (2) S; (3) E+S; (4) E+S+Zn^2+^; (5) E+S+ZnO in pH 7.4 buffer; (6) E+S+ZnO in pH 5.0 buffer and (7) E+S in in pH 5.0 buffer.

**Figure 3:**
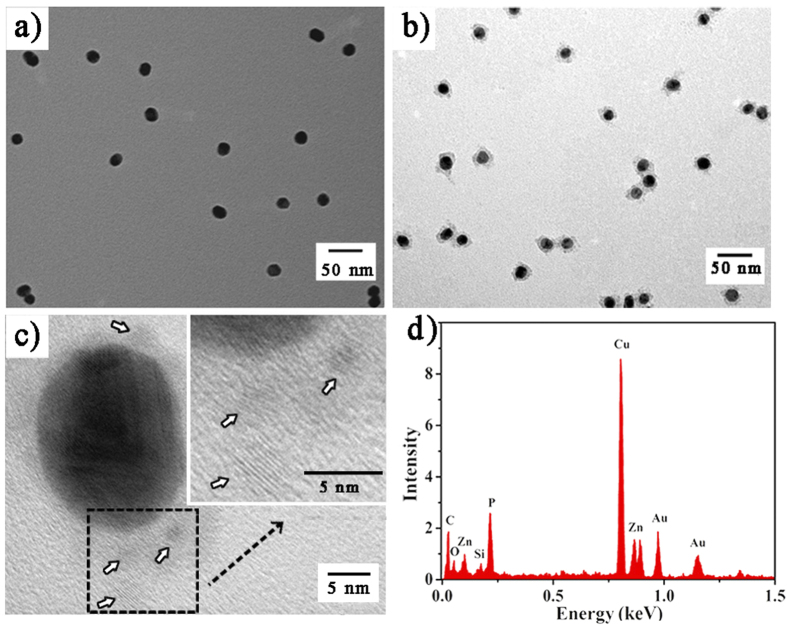
Typical TEM images of (**a**) GNPs-ES and (**b**) GNPs-ES/ZnO. (**c**) HRTEM image of GNPs-ES/ZnO. Inset is the magnified image. (**d**) EDX spectra of GNPs-ES/ZnO.

Figure 1 illustrated the fabrication of the GNPs-ES-Dox/ZnO nanosystem. In the design, water-dispersed GNPs were selected as substrates for DNA conjugation. The morphology of GNPs was revealed by transmission electron microscopy (TEM) as shown in Supplementary Fig. S1. GNPs exhibited a narrow size distribution of 17.6 ± 1.3 nm which was in good agreement with the dynamic light scattering (DLS) result as shown in the inset of Fig. 2a. Thiol-terminated DNAzyme strand, along with nucleolin targeting AS1411 aptamer, was anchored onto GNPs through Au-S bonding, followed by hybridization with its substrate strand containing a ribonucleo-base to yield GNPs functionalized with the hybrid of DNAzyme and substrate (GNPs-ES). The surface functionalization was characterized by UV-Vis, DLS and zeta potential measurements. As displayed in Fig. 2a, the increase in hydrodynamic diameter proved the successful assembly of DNAzyme-functionalized GNPs (GNPs-E). Besides, red shift in UV-Vis spectra which was indicative of increased local refractive index at the surface of GNPs further convinced that DNAzyme had been conjugated to GNPs. According to the fluorescence result, a high surface coverage of ~150 DNAzyme strands per particle was achieved by gradually increasing the concentration of sodium chloride to 0.1 M, which further facilitated the high substrate loading with a density of 100 copies (see Supplementary Information). Such compact payload of DNAzyme hybrid made the resulting GNPs-ES a good carrier for Dox loading. To verify the drug loading capacity, we conducted binding studies between Dox and DNAzyme hybrid. As reported, several anthracycline anticancer drugs including Dox can preferentially intercalate into double-stranded GC pairs, resulting in effective quenching of Dox fluorescence due to the fluorescence resonance energy transfer (FRET) between the intercalated Dox molecules^31^,^32^. With the increase of the DNA duplex concentration, the fluorescence intensity of Dox showed a consecutive reduction (Fig. 2c), which implied the effective loading of Dox. Finally, positively charged ZnO QDs were introduced onto the resultant GNPs-ES-Dox via electrostatic interaction. Amino-functionalized ZnO QDs with a dimension of 3–4 nm were characterized by HRTEM and FTIR spectroscopy as shown in Supplementary Fig. S1. The sensitive response of ZnO QDs to pH value was verified by fluorescence spectroscopy. As shown in Supplementary Fig. S1, high luminescence in neutral condition (pH 7.4) was emitted, while in acidic condition (pH 5.0) the luminescence disappeared rapidly, proving that ZnO QDs were ideal potential Zn^2+^ providers for DNAzyme catalysis. TEM and energy-dispersive X-ray (EDX) analyses were adopted to testify ZnO QDs coating on GNPs-ES. In the case of uncapped GNPs-ES (Fig. 3a), GNPs-ES exhibited similar TEM image with that of GNPs (Supplementary Fig. S1). In contrast, the TEM image of GNPs-ES/ZnO (Fig. 3b) showed that GNPs-ES were surrounded by brighter regions, representing the ZnO QDs coating. In the high-resolution TEM (HRTEM) images (Fig. 3c), ZnO QDs were clearly visible as crystalline spots on the surface of GNPs-ES, suggesting the successful incorporation of ZnO QDs. Moreover, according to EDX analysis in Fig. 3d, characteristic peaks of zinc, silicon and gold further demonstrated the coexistence of ZnO QDs and GNPs in the nanocomposite.

### pH-triggered Activation of DNAzyme

To check the cleavage activity of DNAzyme, polyacrylamide gel electrophoresis was used to separate the cleaved products. As observed in Fig. 2d, the single band in lane 3 suggested that the DNAzyme and its substrate strand formed stable inactive DNA hybrid. With the addition of Zn^2+^, scattering bands appeared in lane 4 owing to the Zn^2+^-triggered DNAzyme cleavage and the subsequent dissociation of the DNA hybrid. The blue arrow corresponded to the cleaved strand, and the green one to the hybrid of DNAzyme and the remaining part. In the cases of lane 5 and 6, ZnO QDs was introduced as the candidate Zn^2+^ source. Typically, in lane 5 (pH 7.4), the cleavage of DNAzyme was blocked due to the absence of Zn^2+^. In comparison, obvious cleaved band was observed in lane 6 (pH 5.0), implying that the dissolution of ZnO QDs in acidic environment leaded to the activation of DNAzyme. Moreover, as a control, lane 7 in acidic environment but without ZnO QDs addition, exhibited less obvious cleavage activity. Combing the above results, we found that the release of Zn^2+^ from ZnO QDs played a pivotal role in the activation of DNAzyme.

### Cellular Uptake of GNPs-ES-Dox/ZnO Probes

**Figure 4:**
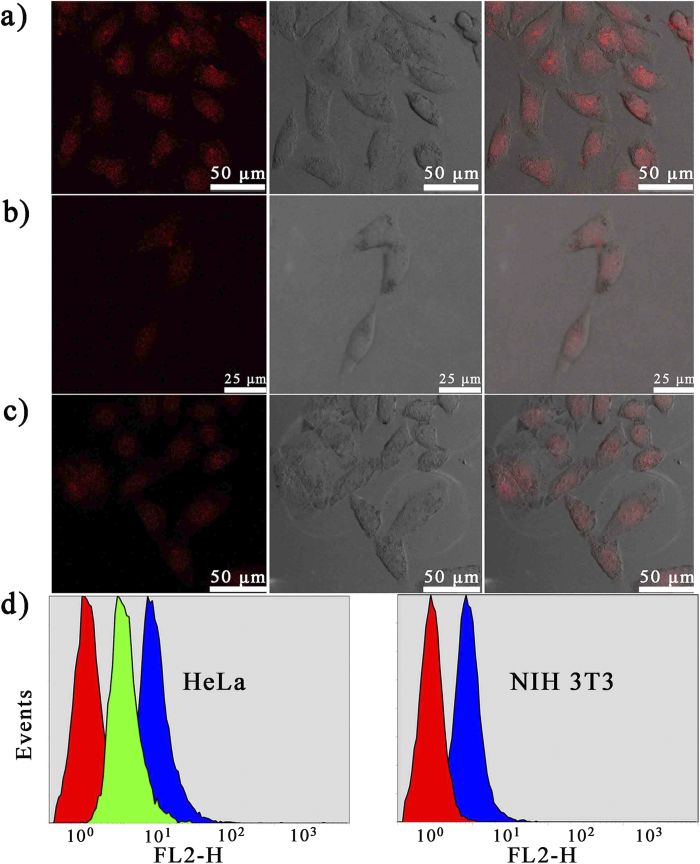
CLSM images of (**a**) HeLa cells and (**b**) NIH 3T3 cells incubated with inactive GNPs-ES-Dox. (**c**) The inhibition assay. The inhibition experiment was conducted by incubating HeLa cells with AS1411 aptamer to block the nucleolin prior to the treatment with inactive GNPs-ES-Dox. (**d**) Flow cytometry analysis of HeLa cells and NIH 3T3 cells incubated with various nanoparticles. For HeLa cells (left to right): control; inhibition assay and inactive GNPs-ES-Dox. For NIH 3T3 cells (left to right): control and inactive GNPs-ES-Dox. The incubation time was 2 h.

**Figure 5:**
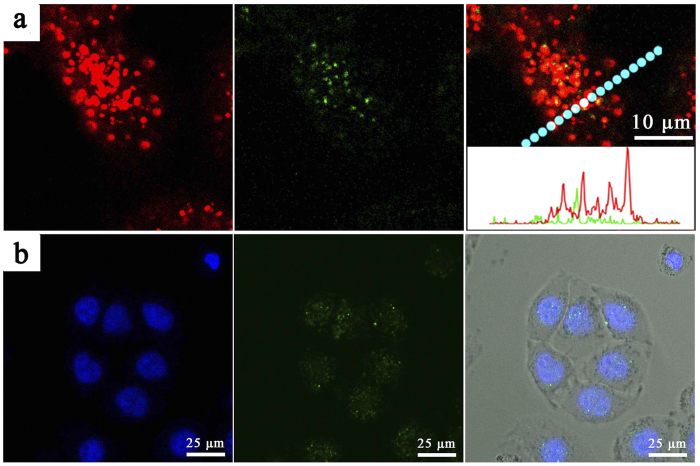
CLSM image of HeLa cells treated by inactive GNPs-ES in which 5′ end of substrate stand was fluorescence labeled with FAM. (**a**) The blue fluorescence from Hoechst 33342 revealed the location of cell nuclei while the green color from FAM indicated the distribution of GNPs-ES. (**b**) The red color came from endo-/lysosomes stained by LysoTracker Red while the green color from FAM. Inset is the line-scan profiles of fluorescence intensity variation for HeLa cells along the marked line.

Prior to further cellular application, *in vitro* cytotoxicity of the nanocarriers was evaluated by MTT assay. Herein, we used an inactive DNAzyme substrate strand, in which the adenosine ribonucleotide was replaced by a deoxyribonucleotide. As shown in Supplementary Fig. S2, with the concentration ranging from 0 to 3 nM, inactive ES-GNPs and inactive GNPs-ES/ZnO displayed similar cellular viability, and no apparent *in vitro* cytotoxicity against HeLa cells was observed, suggesting the good biocompatibility of the nanocarriers. As designed, with the cell-specific recognition function of AS1411 aptamer, the cellular targeting efficiency was anticipated to be greatly improved. The hypothesis was verified by confocal laser scanning microscopy (CLSM) and flow cytometry analysis against the nucleolin-positive HeLa cells and nucleolin-negative NIH 3T3 cells. As shown in Fig. 4, after incubation with inactive GNPs-ES-Dox, much more intensive Dox fluorescence signals was observed in HeLa cells than in NIH 3T3 cells. In the inhibition experiment, HeLa cells which were pre-incubated with AS1411 aptamer for 30 min to block the nucleolin exhibited a weak fluorescence signal. It could be explained that the inhibition of nucleolin would significantly reduce the targeting efficiency, thereby limiting the internalization efficiency. A similar trend was also observed in the results of flow cytometry analysis. As displayed in Fig. 4d, after 2 h incubation with inactive GNPs-ES-Dox, the Dox mean fluorescence intensity of HeLa cells was approximately 2.9-fold higher than that of HeLa cells which were pretreated with free AS1411 to block the nucleolin. Moreover, both cell lines took up inactive GNPs-ES-Dox, while the uptake was 4-fold enhanced in HeLa cells than in NIH 3T3 cells. The results further demonstrated that by virtue of nucleolin-specific aptamer, the cellular targeting and internalization efficiency was remarkably enhanced. In order to further visualize the subcellular distribution of the probes, an inactive DNAzyme substrate whose 5′ end was labeled with FAM was employed. The long distance (>10 nm) between the GNPs and FAM resulted in weak FRET efficiency, and thus making the FAM dye quite suitable candidate for tracing the intracellular localization of the probes. As displayed in CLSM images in Fig. 5, the poor correlation of green (GNPs-ES-FAM) and red fluorescence (endosomes stained by LysoTracker Red) provided direct evidence for the successful escape of the nanoprobes from endosomes. Additionally, a majority of FAM-labeled nanostructures overlapped with the Hoechst 33342-stained nuclei, which clearly revealed that the probes were distributed throughout the HeLa cells, primary in nuclei and less in cytoplasm.

### *In Vitro* Dox Release and Gene Regulation against HeLa Cells

**Figure 6:**
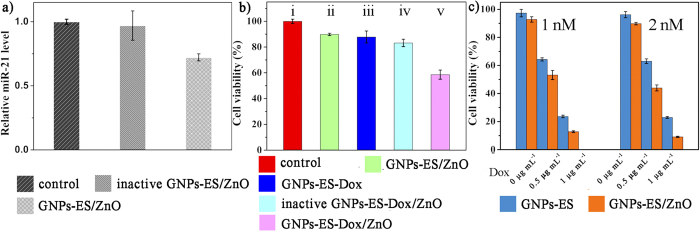
(**a**) The miR-21 expression levels. (**b**) HeLa cell viability after exposure to different nanocarriers at a concentration of 2 nM for 48 h. (**c**) Different concentrations of Dox stimulation on HeLa cells which were pretreated with GNPs-ES or GNPs-ES/ZnO at 1 nM or 2 nM, respectively. Error bars indicate s.d. (n = 3).

The combined treatment of anti-miRNA and chemotherapeutic agent can synergistically increase the anticancer effect, thus providing a promising strategy for cancer therapy^33^. Compared with GNPs-ES, GNPs-ES/ZnO had more potent capability in down-regulating miR-21 as shown in Fig. 6a, highlighting the vital role of ZnO QDs as Zn^2+^ providers to activate cleavage activity of DNAzyme and make a great contribution to miR-21 suppression. Then the therapeutic effect was explored by a series of MTT analysis. As shown in Fig. 6b, GNPs-ES-Dox/ZnO exhibited higher anti-tumor activity than GNPs-ES-Dox, emphasizing the importance of ZnO QDs in driving down miR-21, which gave rise to improved treatment effect. Besides, compared with group ii and iv, the group of GNPs-ES-Dox/ZnO displayed obvious enhanced cytotoxicity, indicating the synergistic effect of gene and drug in inducing cell apoptosis. In order to further demonstrate the phenomenon, we performed the experiments by incubating HeLa cells with GNPs-ES/ZnO or GNPs-ES for 24 h, respectively, and subsequently with different concentrations of Dox for another 24 h. As shown in Fig. 6c, GNPs-ES/ZnO demonstrated slightly higher growth inhibition to the tumor cells in comparison with the control group of GNPs-ES. Whereas, upon stimulation by external Dox, remarkably higher cytotoxic effect was observed in the case of GNPs-ES/ZnO, which was ascribed to the fact that the miR-21 silencing was beneficial to enhancing the sensitivity of HeLa cells to drug, consequently boosting the Dox mediated anticancer effects. Conclusively, combination of chemotherapy and miR-21 silencing made the DNAzyme-conjugated nanosystem an attractive candidate for antitumor therapeutic application.

## Conclusion

In summary, a targeted GNPs-ES-Dox/ZnO nanoprobe capable of co-delivering antisense miRNA and anti-cancer drug was proposed for the combination of gene regulation and drug release. As the gene/drug loading component, DNAzyme/substrate bioconjugate included anti-miR-21 sequence while accommodating Dox molecules. In the composite probe, ZnO QDs were delicately designed as the built-in Zn^2+^ arsenal to activate Zn^2+^-dependent DNAzyme under acidic environment and initiate the following gene/drug release. Such nanocarriers with pH-sensitive behavior have demonstrated that the enhanced therapeutic efficacy can be obtained by suppressing endogenous miR-21 level and synergistically inducing cell apoptosis. Taken together, the integration of anti-cancer drug delivery and selective miRNA regulation makes the probe a potent candidate for multidrug-resistant cancer therapy and *in vivo* application.

## Methods

### Chemicals and Materials

Hydrogen tetrachloroaurate trihydrate (HAuCl_4_), trisodium citrate, L-ascorbic acid, 6-mercaptohexan-1-ol (MCH), zinc acetate, magnesium chloride, 3-aminopropyltriethoxysilane (APTES), tris (2-carboxyethyl) phosphine hydrochloride (TCEP) were purchased from Sigma-Aldrich (St. Louis, MO, USA). 3-(4,5-Dimethylthiazol-2-yl)-2,5-diphenyltetrazolium bromide (MTT) was purchased from Sunshine Biotech. Co. Ltd. (Nanjing, China). The ribonucleo-base-contained substrate stands were obtained from Takara Biotechnology Co. Ltd. (Dalian, China). Oligonucleotide strands were obtained from Shanghai Sangon Biological Engineering Technology & Services Co. (Shanghai, China). The UltraPower^*TM*^ DNA dye was purchased from BioTeke Co. (Beijing, China). LysoTracker Red was bought from Beyotime Institute of Biotechnology (Nantong, China).

The sequences of oligonucleotides (from 5′ to 3′) utilized to assemble the probe are as follows:

DNAzyme strand: SH-TTTTTCTTCTTCTTCTATGTCTCCGAGCCGGTCGAAATAGCTTAT

Substrate strand: TCAACATCAGTCTGATAAGCTAT (rA) GGACATAGAAGAAGAAG

Inactive substrate strand: TCAACATCAGTCTGATAAGCTAT (A) GGACATAGAAGAAGAAG

AS1411 aptamer: TTTTTGGTGGTGGTGGTTGTGGTGGTGGTGG

The underlined bases of the active substrate strand stand for anti-miR-21 which could hybridize with the intracellular miR-21.

### Apparatus and Characterization

UV-Vis spectra were determined using a UV-3600 spectrophotometer (Shimadzu, Japan). Fluorescence spectra for quantification of the DNAzyme loading were recorded on a RF-5301PC spectroflurophotometer (Shimadzu). Other fluorescence determination and MTT assay were performed with the use of a Varioskan Flash (Thermo Scientific, USA). FTIR spectra were collected on a Nicolet 6700 FT-IR spectrometer. DLS measurements were performed on a 90 Plus Nanoparticle Size Analyzer (Brookhaven, USA). CLSM images were obtained using a Leica TCS SP5 confocal microscope. Fluorescence gel imaging was conducted on a Bio-Rad imaging system (serial no. 76S/06725). TEM images were acquired using a JEM 200CX transmission electron microscope (JEOL, Japan) with an accelerating voltage of 200 kV. HRTEM images and EDX measurements were carried out on a JEOL-2100 transmission electron microscope (JEOL, Japan) coupled with an EDX spectrometer. Quantitative RT-PCR (qRT-PCR) assay was conducted on an ABI 7300 Sequence Detection System.

### Preparation of GNPs

GNPs were synthesized following a seeded-growth method^34^. Typically, GNPs with the size of 13 nm as seed liquid were first prepared. 4 mL of HAuCl_4_ solution (25 mM) and 96 mL of ultrapure water were mixed in a 250 mL beaker. The mixture was heated to boiling, followed by the injection of 10 mL of trisodium citrate (1% w/v). The solution color was observed to change from colorless to deep red within several minutes. The system was kept heating for another 20 min and then allowed to cool down slowly under gentle stirring and finally the GNPs were obtained. In the following, 100 mL of the as-prepared GNPs (2 nM) was added into a 250 mL beaker. 1.96 mL of trisodium citrate (1% w/v), 1.96 mL of HAuCl_4_ aqueous solution (24.2 mM) and 1.96 mL of L-ascorbic acid (1% w/v) were added at an interval of 1 h, respectively. Afterwards, the solution was heated to keep at the boil for 30 min, and cooled down slowly. Eventually, the GNPs were prepared. The extinction coefficient of the resulting GNPs is 1.13×10^9^ M^−1^cm^−1^ at 520 nm.

### Synthesis of Amine-Functionalized ZnO QDs

Amine-capped ZnO QDs were prepared following the reported procedures with slight modifications^27^. 440 mg of Zinc acetate and 44 mg of magnesium chloride were dissolved in 30 mL of hot ethanol under stirring. In another flask, 100 mg of NaOH (2.5 mmol) in ethanol was heated to reflux. Both of the solutions were quickly cooled down in an ice bath. Then the NaOH solution was fleetly injected into the flask containing zinc acetate and magnesium acetate. The mixture was kept stirring for 8 h. Afterwards, the as-fabricated nanoparticles were precipitated using hexane and centrifuged at 1000 rpm for 5 min. The pellets were dispersed in 15 mL of N, N-dimethylformamide (DMF) by ultrasound and heated to 120 °C, followed by adding 40 μL of APTES under vigorous stirring. The mixture was continuously stirred at the temperature for another 15 min. Then the products, amine-capped ZnO QDs, were collected by centrifugation at 12,000 rpm for 8 min, washed with DMF, and finally redispersed in 1.5 mL water.

### Conjugation of DNAzyme on GNPs

Typically, to break up disulfide linkage of thiolated DNAzyme strand, 500 μL of DNAzyme (10 μM), 100 μL of TCEP (0.03 mg mL^−1^) and 1 μL of NaAc-HAc buffer (500 mM, pH 5.2) were mixed together and kept for 1 h in the dark. The reduced DNAzyme was then added into 4.35 mL of GNPs (5 nM) solution. The mixture was shaken overnight at a gentle speed of 100 rpm. Afterwards, Tris-HAc (500 mM, pH 8.2) was brought in to achieve a 20 mM Tris-HAc concentration. Subsequently, salt-aging was conducted by slowly increasing the salt concentration to 0.1 M. After standing for another 24 h, the resultant GNPs-E solution was stored at 4 °C for use.

### Hybridization with Substrate Strand

The GNPs-E solution was purified by twice centrifugation, and redispersed in 20 mM HEPES (pH 7.4, 100 mM NaCl) which was prepared using DEPC treated ultrapure water. Then substrate with a ratio of 230 strands per particle was added to the GNPs-E solution. The mixture was maintained at 65 °C for 5 min and cooled down slowly at room temperature for 1 h, after that, stored at 4 °C for 12 h to achieve full hybridization. The product GNPs-ES was isolated by centrifugation, then washed three times with 20 mM HEPES buffer (100 mM NaCl) and finally dispersed in the HEPES buffer for use.

### Intercalation of Dox and Cladding with ZnO QDs

The GNPs-ES was incubated with 10 μM Dox at room temperature. After 20 min, the solution was washed four times by centrifugation to remove the residual Dox. To prove the intercalation of Dox, we fixed the concentration of Dox, and then added a series of different concentrations of hybridized equivalent of DNAzyme and substrate ranging from 0 to 1 μM. The fluorescence intensity was measured using a microplate reader.

To clad the probe with ZnO QDs, GNPs-ES-Dox (2.5 nM) solution in 10 mM HEPES buffer was incubated with 0.03% (v/v) ZnO QDs, shaken at room temperature with a speed of 180 rpm for 30 min. The solution was then centrifuged and washed with 20 mM HEPES buffer for three times. The assembly with ZnO QDs was confirmed by TEM and EDX analysis.

### Gel Electrophoresis Analysis

8% polyacrylamide gel was employed to examine the cleavage activity of DNAzyme. DNAzyme and substrate strand were incubated in 20 mM HEPES buffer (100 mM NaCl) for 3 h (final concentration: [DNAzyme] = [substrate] = 1 μM) to yield inactive DNA hybrid. Then Zn(Ac)_2_, ZnO QDs, HEPES (pH 7.4) and MES (pH 5.0) were added to the corresponding solution, respectively (final concentration: [Zn(Ac)_2_] = 10 mM, [ZnO QDs] = 5% (v/v), [HEPES] = 50 mM, [MES] = 50 mM). All the mixtures were kept at room temperature for 45 min. Finally, the samples were pre-stained by UltraPower^*TM*^ DNA dye following the instructions provided by the manufacturer and then loaded into polyacrylamide gel. The electrophoresis analysis was conducted at 100 V for 50 min and recorded by the fluorescence gel imaging system.

### miR-21 Detection by qRT-PCR

For estimation of the miR-21 level, the oligonucleotides used were listed as following:

miR-21 sense primer: 5′-ACACTCCAGCTGGGTAGCTTATCAGACTGA-3′

miR-21 antisense primer: 5′-CTCAACTGGTGTCGTGGAGTCGGCAATTCAGTTGAGTCAACATC-3′

miR-21 reverse primer: 5′-CTCAACTGGTGTCGTGGAGTCGGCAATTCAGTTGAGTCAACATC-3′

U6 sense primer: 5′-CTCGCTTCGGCAGCACA-3′

U6 antisense primer: 5′-AACGCTTCACGAATTTGCGT-3′

U6 reverse primer: 5′-AACGCTTCACGAATTTGCGT-3′

U6 was used as internal control to normalize the miR-21 expression levels. The relative amounts of miR-21 were calculated using the comparative threshold cycle method as 2^−ΔCT^, where the ΔCT = CT (experimental miR-21) – CT (U6).

### Cell Viability Analysis

NIH 3T3 mouse fibroblast cells and human cervical carcinoma cells (HeLa cells) were purchased from Nanjing KeyGen Biotech Co. Ltd. and seeded in complete DMEM (KeyGen Biotech Co. Ltd.) containing 10% penicillin (80 U mL^−1^), streptomycin (0.08 mg mL^−1^) and 10% fetal calf serum at 37 °C in a 5% CO_2_ incubator. Cell viability was assessed by MTT assay. In brief, 5000 cells per well in 100 μL medium were seeded in 96-well plates. After incubation for 24 h, the medium was removed and replaced with fresh medium containing samples of different concentrations. Particularly for the experiment of Dox stimulation, after incubation with specific nanoparticles for 24 h, the cells were then cultured with various concentrations of Dox (from 0 μg mL^−1^ to 1 μg mL^−1^) in fresh medium for another 24 h. At the corresponding time points, the medium was substituted with 100 μL MTT-containing medium (final concentration: [MTT] = 0.5 mg mL^−1^). After 4 h incubation under 5% CO_2_ atmosphere at 37 °C, dimethyl sulfoxide (DMSO, 100 μL) was added to each well to replace the MTT-containing medium. The optical absorbance at 490 nm was measured by a Varioskan Flash.

### Fluorescence Tracking of the Internalization of the Probe

After culturing HeLa cells and NIH 3T3 cells for 24 h, 1 mL of medium containing 2 nM inactive GNPs-ES-Dox or 2 nM inactive GNPs-ES in which the substrate strand was fluorescence labeled with FAM, was added to substitute the medium. After 2 h incubation under 5% CO_2_ at 37 °C, the cells were washed three times with sterile PBS. Then CLSM imaging was performed with a confocal microscope.

## Additional Information

**How to cite this article**: He, Z.-M. *et al.* A Targeted DNAzyme-Nanocomposite Probe Equipped with Built-in Zn^2+^ Arsenal for Combined Treatment of Gene Regulation and Drug Delivery. *Sci. Rep.* **6**, 22737; doi: 10.1038/srep22737 (2016).

## Supplementary Material

Supplementary Information
