# Supplementary material for: A Targeted DNAzyme-Nanocomposite Probe Equipped with Built-in Zn2+ Arsenal for Combined Treatment of Gene Regulation and Drug Delivery
Source: Sci Rep. 2016 Mar 9;6:22737. doi: 10.1038/srep22737 (PMC4783697; doi:10.1038/srep22737)
Supplement: Supplementary Information [file srep22737-s1.pdf]

Supplementary Information

A Targeted DNAzyme-Nanocomposite Probe Equipped with Built-in Zn^{2+} Arsenal for Combined Treatment of Gene Regulation and Drug Delivery

Zhi-Mei He[1], Peng-Hui Zhang[1], Xin Li[2], Jian-Rong Zhang[1,3,*], and Jun-Jie Zhu[1,*]

[1]State Key Laboratory of Analytical Chemistry for Life Science, School of Chemistry & Chemical Engineering, Nanjing University, Nanjing 210093, China

[2]State Key Laboratory of Pharmaceutical Biotechnology, School of Life Science, Nanjing University, Nanjing 210023, China

[3]School of Chemistry and Life Science, Nanjing University Jinling College, Nanjing 210089, China

*Correspondence and requests for materials should be addressed to Z.J.J. (email: jjzhu@nju.edu.cn or Z.J.R. (email: jrzhang@nju.edu.cn)

The Supplementary Information includes:
-Additional Experimental Section
-Supplementary Figures S1 and S2

Additional Experimental Section

Calculation of DNAzyme Loading on GNPs. The number of DNAzyme strands per nanoparticle was calculated by the fluorescence intensity of FAM labeled at the 3' ends of the strand. The quantification was accomplished by two methods. First, we quantified the conjugated DNAzyme by the fluorescence of FAM with or without GNPs that was completed by collecting the unconjugated DNAzyme after centrifugation. The second method was performed by displacing the surface-coordinated DNAzyme through MCH treatment. Briefly, we incubated the GNPs-E nanoconjugation with 1 mM MCH overnight. Then, the displaced DNAzyme was separated from the solution by centrifugation. Finally, the concentration of the conjugated DNAzyme was determined by the fluorescence signal of FAM. Both the two methods gave similar estimation that each GNP was loaded with approximately 150 copies of DNAzyme.

Quantification of DNAzyme Substrate Strands Loading. To measure the number of substrate strands which were hybridized with GNPs-E, FAM-labeled substrate stand was selected. The estimation methods were carried out as the calculation of DNAzyme loading. Both of the results showed that a high surface coverage of about 100 substrates per GNP was achieved.

Supplementary Figures

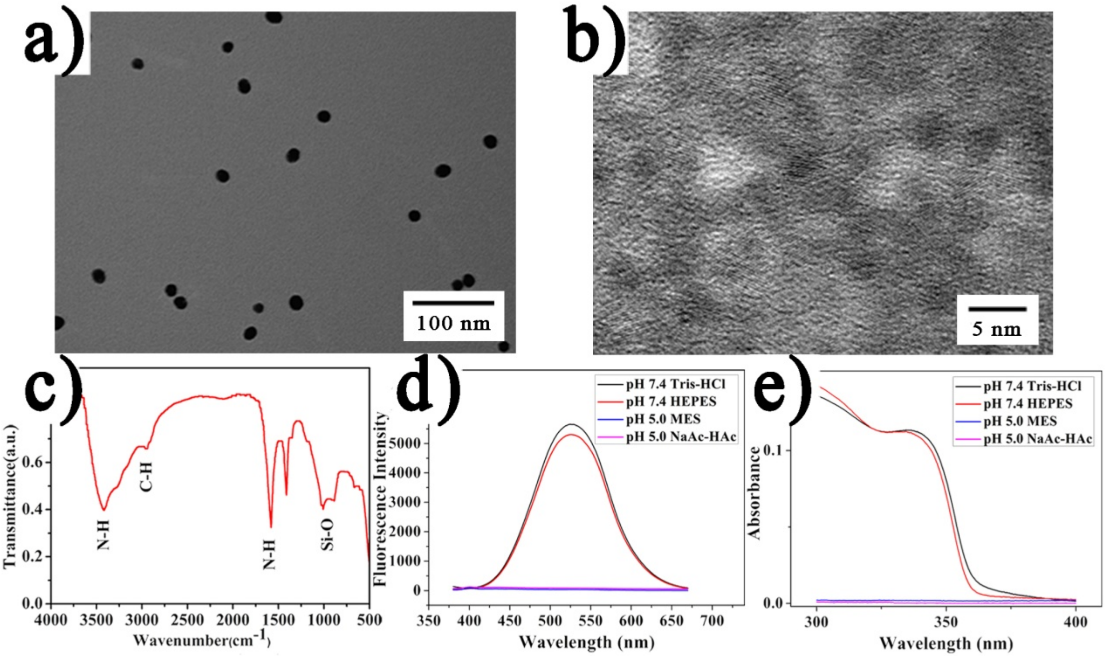

Figure S1. (a) TEM image of GNPs. (b) HRTEM image of ZnO QDs. (c) FTIR spectrum of aminated ZnO QDs. (d) Fluorescence spectra and (e) UV-Vis spectra of ZnO QDs dispersed in 20 mM Tris-HCl (pH 7.4, black), HEPES (pH 7.4, red), MES (pH 5.0, blue) and NaAc-HAc (pH 5.0, purple).

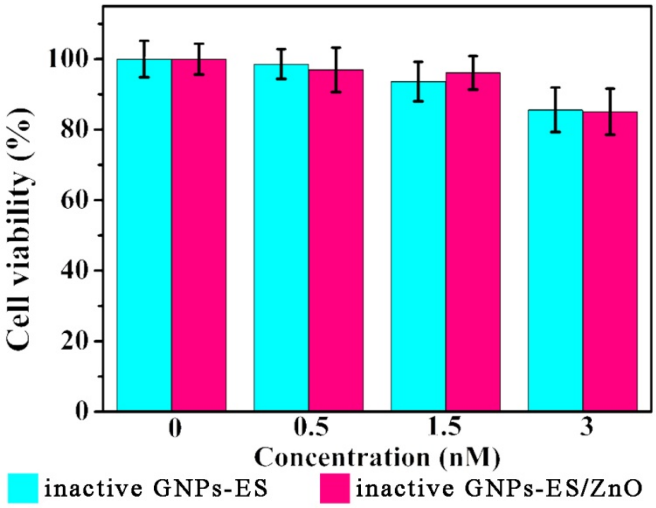

Figure S2. Cell viability of HeLa cells after 24 h exposure to inactive GNPs-ES or inactive GNPs-ES/ZnO.
